# Suicide Prevention Mobile Apps for Indian Users: An Overview

**DOI:** 10.7759/cureus.16770

**Published:** 2021-07-31

**Authors:** Sindhuja Sudarshan, Seema Mehrotra

**Affiliations:** 1 Clinical Psychology, National Institute of Mental Health and Neurosciences, Bengaluru, IND

**Keywords:** suicide prevention, india, suicide, mobile applications, android applications, mhealth

## Abstract

Suicide is an issue of global concern. Mobile applications (apps), if found effective, could supplement suicide prevention efforts by addressing some of the barriers to help-seeking. This study aimed to review the nature of suicide prevention apps available for Indian users using the Android platform. Apps identified were broadly reviewed on general features, inclusion of educative elements, suicide risk assessment, and strategies to manage suicidal thoughts. The search terms “suicide,” “suicide prevention,” and “feeling suicidal” were used to search the Google Play Store from May to December 2020, and apps exclusively focusing on suicide prevention were identified and reviewed.

The initial search resulted in 492 apps, of which 43 met inclusion criteria and were further assessed. Fewer than half the apps included supplementary information to users on scientific, evidence-based content (32.55%), and only a few apps were reported to be empirically validated (11.62%). Approximately one-third of the apps intended for people at suicidal risk had an initial screening aspect (16.12%), and one-third of the apps intended for support providers had a suicide risk assessment tool (25.92%). Most apps (81.39%) included a suicide helpline number specific to the region where they were developed, but only a few (23.25%) included motivational elements to call helplines. Common therapeutic strategies suggested to manage suicidal thoughts included distraction, means restriction, environment safety, perspective-shifting strategies, and calming or soothing strategies. Several apps (39.53%) included therapeutic strategies through safety plans.

For apps to be used effectively for suicide prevention, they should include evidence-based content and motivational elements to call helplines, and and clinicians need to examine app features and content before recommending them for use by patients.

## Introduction and background

Suicide results in thousands of preventable deaths worldwide every year and has been a cause for global concern. Most suicide-related deaths occur in low- and middle-income countries and are estimated to be the highest in the South-East Asia region [1]. At 16.5/100,000, the suicide rates in India stand higher than the global average of 10.5/100,000 [2]. Suicide was reported to be the leading cause of death in India in 15- to 39-year-olds in 2016 [3]. Furthermore, those living in urban metropolitan Indian cities tend to show a greater risk of suicidality than rural areas [4]. Despite these worrying figures, various barriers to help-seeking continue to thwart efforts toward suicide prevention.

To ensure that individuals at risk of suicide receive appropriate care and support, timely identification is crucial [5]. However, most individuals at risk of suicide, especially in low- and middle-income countries, do not seek help due to low perceived need and various structural and attitudinal barriers [6]. This highlights the urgent need to use novel, acceptable, and cost-effective methods to bridge the treatment gap. To increase availability and access to evidence-based mental health care, the World Health Organization’s Mental Health Action Plan recommends using self-help interventions through electronic and mobile technologies besides other measures [7]. Technology has been used to deliver self-help and guided psychological interventions with promising results and could serve as a low-intensity and low-cost strategy to cater to a larger population in need of treatment [8]. Specific to suicide prevention, there is an emerging upward trend in the use of technology for the prevention of suicidal behaviors over the last decade [9]. With the rapid emergence of various technologies aimed at suicide prevention, there is a need for research studies evaluating the benefits and risks of using technology in suicide prevention as also the effectiveness of these technologies in reducing suicidal behaviors so they could be implemented appropriately in clinical practice [9].

The rapid development of technology worldwide has led to the recent emergence of mobile health (mHealth), which can monitor and manage mental health-related concerns through mobile technologies like health-related applications, websites, and text messaging [10]. Factors including low cost, flexibility of access, and discreetness have popularized mental health-related mobile applications (apps) among the public. It is estimated that there are over 10,000 apps on mental health alone, with the number increasing manifold [11].

Internet penetration rates and the use of smartphones are steadily increasing in India. With around 500 million smartphone users, India is among the nations with the highest app downloads [12]. Mobile mental health could be a scalable option for India to effectively aid and supplement mental health services [13]. Considering the ease of access and anonymity that apps offer and the increasing number of young, urban Indians with access to smartphones, apps could be well utilized for suicide prevention.

However, among the massive number of apps potentially available to users, only a small proportion have been empirically validated, making it tedious for users to identify an app suitable for their concerns [5, 14]. Apps also have their share of ethical issues and risks [15-16]. In addition, low mental health literacy and limited to no data on the utility of the information provided within the app adds to the complexity of finding a relevant app [17]. Previous reviews on suicide prevention apps have highlighted the need to scrutinize app content given the lack of guidelines in app stores about the restriction of pro-suicidal content [18]. Further, a recent systematic review evaluating the effectiveness of suicide prevention apps found that mobile apps were effective in reducing the risk of suicide and improving adaptive skills. However, the authors found that only a few apps were designed to reduce suicidality and assessed effectiveness using quality methods [19]. Since suicide prevention apps could be accessed during a crisis, it is also crucial that apps provide brief and relevant information and access to appropriate helplines [20]. Given this, we reviewed the nature of apps addressing suicide prevention available for Indian users on the Android platform, considering that most smartphone users in India use an Android device [21]. Specifically, the review examines the general features of the app, the inclusion of educative elements and assessment of suicide risk, and the strategies to manage suicidal thoughts incorporated in these apps.

## Review

The search terms “suicide,” “suicide prevention,” and “feeling suicidal” were used to search the Android store (i.e., the Google Play Store) from May 2020 to December 2020. All apps available for free, in English, for Indian users were examined for their relevance to the present study. Further, the list of apps generated was verified with the Google Play Store on a web browser to ensure thoroughness. The initial search resulted in approximately 492 apps, of which only 137 were found to focus purely on suicide prevention or addressed suicide as one of the components. Most of the remaining 355 apps were found to be games that included the terms “suicide” or “dead/death” (n=92), quotes or wallpapers on sadness or death (n=107), apps for other mental health disorders such as stress, anxiety, and panic or apps on meditation and relaxation (n=156), and these were excluded from the review.

**Figure 1:**
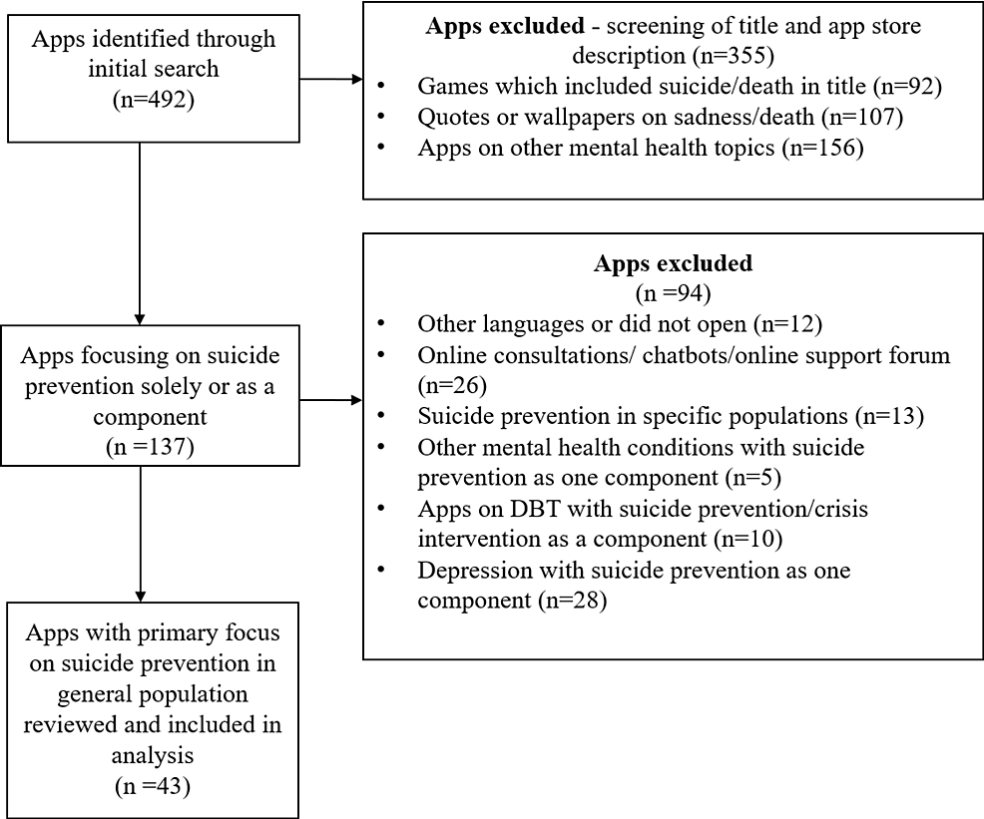
App selection flowchart. DBT, dialectical behavior therapy

The remaining 137 apps were downloaded and further assessed for suitability. The study excluded apps that did not open or were in languages other than English (n=12) and those involving chatbots or online consultations where the content was either unavailable for scrutiny or had no interactive material specifically addressing suicide prevention (n=26). Apps on suicide prevention intended for specific populations such as the defense services or participants in specific suicide prevention programs (n=13), and those intended for other mental health conditions with suicide prevention as only one component (n=5) were excluded. Other eliminations included apps focusing on dialectical behavior therapy with suicide prevention/crisis intervention (n=10) and those primarily addressing depression with suicide prevention (n=28). Thus, a total of 43 apps focusing exclusively on suicide prevention were reviewed further (Figure 1).

A structured data sheet was prepared for the present review by both authors based on previous studies in the area [5,18] and apps were subsequently rated on each parameter as present/absent by the first author and discussed subsequently with the second author. The apps were examined in terms of the following parameters: a) registration/password protection to access content, for better security; b) presence of a privacy policy or user agreement within the app that includes information about the privacy and extent of confidentiality of the data obtained; c) disclaimer statements clearly stating the limits of the app and not intended as substitute for professional advice and the importance of seeking professional help when needed; d) clarity regarding the target audience (whether the app was targeted at individuals with suicidal ideation, support providers, or had relevant content for both audiences); e) explanation regarding the scope of the app within the app clearly stating the objectives and purpose of the program; f) provision of evidence-based information in the app to help users make informed choices; g) educative content about suicide and suicide prevention; h) provision of an initial screening for those contemplating suicide and suicide risk assessment as well as guidance for support providers; i) provision of suicide helpline numbers, list of emergency services, and local community resources for those actively contemplating suicide or for support providers and motivational elements to promote help-seeking; j) provision of tips/strategies to deal with suicidal thoughts.

Some features were standard across all apps, while others were relevant for particular audiences. For example, apps intended for support providers of those at suicidal risk were examined for the presence of suicide risk assessment and feedback guidelines for the course of action. Similarly, we assessed the apps intended for those at risk of suicide to determine if the apps offered an option for developing a safety plan or provided strategies to deal with suicidal thoughts.

General features

All apps were free to download with one app (2.32%), providing limited content free of charge and full content as a paid feature/in-app purchase. One app (2.32%) included an option for voluntary donations. Apps addressing issues such as suicide prevention are likely to be accessed by individuals at times of crisis when their thinking and functioning may be compromised. Therefore, such apps must provide brief and easily accessible information [20]. Considering this, most apps (n=37; 86.04%) had a simple menu to navigate, and 35 apps (81.39%) had brief and easy to comprehend content.

Table 1 summarizes the general features of the apps reviewed. Concerning features regarding privacy and confidentiality, only one app (2.32%) mandatorily required users to register to gain access to app content. Fewer than half the apps (19 of 43; 44.18%) provided a privacy statement or terms of use within the app explaining the use of data being collected and limits to confidentiality. This is vital as users may provide sensitive information, often unaware of its use by the developer [17]. Two apps collected personal information like phone numbers without a privacy policy. While gathering personal information such as name and contact details may be pertinent in aspects such as suicide prevention, it is also essential that users know the limits of confidentiality and the potential use of their data. Therefore, features such as password protection are essential [20]. Further, deficiencies in password protection and a lack of privacy policies could deter consumers and professionals from relying on apps and reduce their trustworthiness [18, 22]. Twenty-two apps (51.16%) provided a disclaimer that the app was not a substitute for medical advice and explained the apps’ limits.

**Table 1:** Features of apps reviewed (N=43).

Feature	Number of apps (percentage)
Registration mandatory for use	01 (2.32%)
Privacy statement and/or terms of use within the app	19 (44.18%)
Disclaimer stating the limits of the app in not being a substitute for medical advice/care	22 (51.16%)
Clear description of intended audience in initial screens provided within the app (for whom)	28 (65.11%)
Scope explicitly delineated within the app	27 (62.79%)
Mention of app content being empirical/based on research	14 (32.55%)
Specific mention of theoretical framework used as basis for the app	02 (4.65%)
Provides only information and education on suicide but no interactive features	10 (23.25%)

Of the 43 apps reviewed, 15 apps (34.88%) were targeted directly to those at risk of suicide, 15 apps (34.88%) to those at suicidal risk and their support system, 12 apps (27.9%) at support providers, and one app (2.32%) at individuals bereaved by suicide (Table 2).

**Table 2:** Intended audience for suicide prevention apps reviewed (N=43).

Intended audience	Number of apps (percentage)
Individuals at suicidal risk	15 (34.88%)
Individuals at suicidal risk and support providers	15 (34.88%)
Support providers of person at suicidal risk	12 (27.9%)
Individuals bereaved by suicide	01 (2.32%)

Guidelines developed for technology-based suicide prevention programs highlight the importance of clearly specifying the target population [22]. Only 28 apps (65.11%) stated their intended audience clearly (Table 1). As suicide prevention apps may be accessed at a time of crisis, it would be helpful for apps to explicitly state the intended audience to enable users to access appropriate sections quickly and efficiently.

Apart from clarity on the intended audience, apps should provide a clear overview of their objective, purpose, and contents. This could help users make informed choices and choose a relevant app with greater ease. Approximately 27 apps (62.79%) explained the scope of the app clearly and explicitly (Table 1).

Approximately 25 apps (58.13%) provided information regarding developers within the apps. In most cases, developers were either government organizations, private companies, or those engaged in mental healthcare in general or suicide prevention specifically. This finding is similar to previous studies reviewing suicide prevention apps [5, 18]. While information regarding developers could not be found within the app or on the developer’s website for six of the apps reviewed (13.95%), 13 apps (30.23%) were developed by governments or in collaboration with the government, and 10 apps (23.25%) were developed by private companies. Among the private companies, five companies provided information on mental health experts on the development team. Among the remaining apps, six (13.95%) were developed by mental health professionals and eight (18.6%) by not-for-profit organizations. Despite most apps being developed by organizations and institutes involved in mental healthcare, only five (11.62%) provided information about a formal evaluation process, such as research studies evaluating the app’s effectiveness. Reviews examining the effectiveness of online and mobile health technologies for suicide prevention suggest that they may show some effectiveness for self-management; however, considering that evaluative research lags far behind the pace of app development, evidence is preliminary and insufficient [23-24]. The American Psychiatric Association also recommends examining end-user feedback, evidence of benefit from trusted sources such as academic institutions, or relevant research studies before recommending an app for use [25]. Among other aspects, having a formal evaluation process in place also helps improve an app’s credibility beyond user ratings alone [20]. 

Further, 14 apps (32.55%) explicitly mentioned providing information/strategies informed by research, and only two apps (4.65%) specifically mentioned the school of therapy or strategies provided in the app for managing suicidal risk. Twenty-eight apps (65.11%) provided references or online resources for the app’s content or further information. By not including appropriate references for content, it would be difficult for potential users to determine the credibility of the information provided [18, 20]. Only four of the 43 apps reviewed were developed in India. Overly simplistic content and lack of clarity about a professionally recognized helpline number were noted in one of these apps.

Information regarding suicide and assessment of suicidal risk

Ten apps (23.25%) provided information about suicide prevention and lacked interactive features or strategies for dealing with suicidal thoughts. Here, the information provided was through text or videos. It included sections on educating support providers about suicide, its causes, myths regarding suicide (n=20; 46.51%), and warning signs (n=32; 74.41%), but often did not have a separate section on crisis management or strategies to deal with suicidal thoughts (Table 3). This finding supports previous reviews on suicide prevention apps [5]. Of the 27 apps aimed at support providers, 20 (74.07%) had tips on broaching the topic of suicide with those at suicidal risk.

**Table 3:** Nature of information provided by apps (N=43). *Of 27 total: apps intended for both individuals at suicidal risk and support providers, as well those intended for support providers alone. †Of 31 total: apps intended for individuals at suicidal risk, those at suicidal risk and support providers, as well as for those bereaved by suicide.

Nature of information	Number of apps (percentage)
Educative elements	
Warning signs about suicide	32 (74.41%)
Myths pertaining to suicide	20 (46.51%)
References for content and online resources for further information	28 (65.11%)
Guidance for support persons	
Ways to discuss about suicidality with a person at suicidal risk	20 (74.07%)*
Suicide risk assessment tools in apps targeted at support persons	7 (25.92%)*
Screening and safety plans for those at suicidal risk	
Solely intended for creating a safety plan for individuals at suicidal risk	7 (22.58%)†
Initial screening/evaluation for apps targeted at individuals at suicidal risk	5 (16.12%)†
Helplines/emergency resources	
Provides suicide helpline numbers	35 (81.39%)
Connectivity to suicide helpline provided within app	33 (76.74%)
Helpline number listed on every page of the app	15 (34.88%)
Mental health services/emergency resources in the vicinity	21 (48.83%)
Solely provides suicide helpline number/emergency services and no other information pertaining to suicide prevention	02 (4.65%)

Among the 31 apps targeted to individuals at suicidal risk alone, those bereaved by suicide, and those at risk as well as their support network, only five apps (16.12%) included an initial screening/evaluation (Table 3). These apps typically provided mood rating options; Likert scale questions to assess the degree of suicidality; suicidal thoughts, behaviors, and risk factors to varying extents; and recommended appropriate sections within the app. Among them, only one app included an evidence-based measure of distress, and one included an assessment for suicidal thoughts and behavior.

Only seven of the 27 apps (25.92%) intended for support providers included a suicide risk assessment. Among them, some provided support strategies to help those contemplating suicide based on the level of suicidality identified through the risk assessment.

Suicide prevention strategies incorporated in the apps

Suicide could be a result of various causes and is associated with many factors. Prevention involves the interplay of several strategies. Best practice guidelines for individual factors recommend using awareness and gatekeeper training programs, including education and information regarding suicide, frequent screening in emergency and primary care settings, access to means restriction, pharmacotherapy, psychotherapy, and follow-up care [26]. While several apps considered in this review incorporated educative elements and provided access to helplines, few suggested strategies for managing suicidal thoughts. Table 4 provides an overview of suicide prevention intervention strategies included in the apps.

**Table 4:** Suicide prevention intervention strategies suggested by apps (N=43).

Intervention strategy	Number of apps (percentage)
Explicit suggestion to seek professional help	35 (81.39%)
Creating and committing to safety plan	17 (39.53%)
Tips on dealing with crisis for helper	25 (58.13%)
Distraction	17 (39.53%)
Means restriction and environment safety	16 (37.20%)
Perspective-shifting and cognitive strategies	16 (37.20%)
Calming/soothing strategies	11 (25.58%)
Focusing and problem-solving strategies	07 (16.27%)
Self-care and well-being	07 (16.27%)
Motivational strategies	06 (13.95%)

Of the 27 apps intended for support providers, five (18.51%) included warning signs of suicide as an interactive assessment and provided strategies to help those at suicidal risk according to the risk level. Strategies suggested included showing support by listening and validating distress, checking for suicide plans, removing access to means, encouraging help-seeking from family and friends or professionals, calling a helpline, and staying with the person until help arrives. Some apps also suggested following up and supporting the person after the crisis. Also, one app provided a level of suicidal risk with no further feedback or suggestions. A few apps included a detailed evaluation of suicidality. The Suicide Prevention App [27] assessed suicidal thoughts and behavior and assessed past suicidal attempts, safety concerns, and protective factors. The app provided a report and immediate steps that the helper could take to help the suicidal individual based on the severity level identified on the assessment. In general, 25 apps (58.13%) offered support providers tips/strategies to help an individual at risk of suicide during a crisis.

Support mobilization

Most apps encouraged individuals at risk of suicide/their support network to seek help and support from loved ones, crisis support services, or mental health professionals, most often through the section on safety plans. Support strategies provided by apps included reaching out to close family members/peers for help (35/43; 81.39%), explicit suggestions to seek professional help (35/43; 81.39%), and calling suicide helpline numbers/emergency services during a crisis (32/43; 74.41%). While 35 apps (81.39%) recommended reaching out for support during a crisis, nearly half (20/43; 46.51%) facilitated contact with the concerned person from within the app.

Several apps included the national suicide helpline number (35/43; 81.39%), with 33 (76.74%) providing access and direct connectivity to the helpline from within the app. If suicide prevention apps are developed to improve access and bridge the gap between community and support services, immediate connectivity to crisis helplines from within the app would be essential [20]. Further, listing and ensuring connectivity to a helpline from every app page would be necessary for prompting users to seek immediate help during a crisis [22]. Considering this, fewer apps (15/43, 34.88%) had the helpline number listed on every page within the app. Also, 21 apps (48.83%) listed mental health or emergency services available in the user’s vicinity, with five apps (11.62%) using Global Positioning System data to locate those services.

Motivational enhancement for help-seeking

Only a few apps (10/43; 23.25%) included motivational elements that sought to break barriers in help-seeking beyond one-line suggestions to seek help or call helplines. These apps sought to normalize the anxiety associated with calling a helpline by describing what to expect while calling a helpline or meeting a mental health professional, or the apps provided brief descriptions of common mental health disorders and explained when to seek professional help. Only a few apps included motivational quotes on help-seeking and quoted positive experiences of individuals who had benefited from professional help in dealing with suicidal ideation. Among the apps for support providers, two encouraged users to pledge to listen and help an individual at suicidal risk who sought help and provided support persons with brief information and suggestions on offering help. A study that reviewed apps on depression also found that only a few included content that destigmatized and demystified professional help-seeking [17].

Safety plans

Several suicide prevention apps also incorporated suicide prevention strategies through safety plans. A total of 17 apps (39.53%) either had a separate section on safety plans (10/43; 23.25%) or were intended primarily to help an individual at risk of suicide develop a safety plan (7/31; 22.58%). Some apps solely provided information regarding safety plans (2/17; 11.76%), while a majority included interactive features to help users create safety plans within the app (15/17; 88.23%), with a further five apps providing users options to either e-mail or print the plan from within the app. The apps drew components mainly from Stanley and Brown’s safety planning intervention [28] to differing extents. The safety planning intervention [28] is recognized as a best practice brief intervention [29]. It includes six components: identifying warning signs of a crisis, internal coping strategies, people and places for distraction and support, approaching people for help, contacting mental health professionals, and reducing access to lethal means [29]. Only six of the 17 apps included all the six components of the safety planning intervention. Martinengo et al. also found that only a small proportion of suicide prevention apps included all the steps for developing a safety plan [5].

Regarding the six components, 15 apps included identifying triggers or warning signs of a suicidal crisis. Sixteen apps each included a section on coping strategies and approaching professionals for help in resolving a crisis, with 15 of these apps facilitating access to helplines from within the app. A section on seeking help from family/friends was present in all apps. On restricting access to means, 10 apps included suggestions of securing objects which could be potentially used to harm oneself or going to a safe place.

Apps that included intervention components typically addressed one or more strategies that involved elements of cognitive behavior therapy and dialectical behavior therapy. Both approaches are considered effective in reducing suicidal behavior risk [29-30]. A few apps included stand-alone suggestions or strategies apart from the safety plan. For this review, the suggestions or techniques were grouped into broader strategies discussed below based on the underlying rationale mentioned in the app (Table 4).

Distraction

Distraction was the most suggested strategy, with 17 of 43 apps (39.53%) encouraging users to keep themselves engaged in activities to delay acting on suicidal thoughts. Some suggestions included doing the opposite of how one feels, conversing with loved ones, working on chores and routine activities at home, engaging in meaningful activities such as creating art, joining activity clubs, school clubs or spirituality, or exploring nature. Among the coping strategies suggested, one app included a list of user-suggested activities that individuals at risk of suicide could use during times of crisis. One such suggestion subtly hinted at self-harm, although intending to help the user consider more healthy coping strategies in its place.

Means Restriction and Environment Safety

Several apps (16/43, 37.20%) encouraged users to remove access to means of suicide or to stay away from places with easy access to potentially harmful objects and to identify places for distraction or where they feel safe. Other suggestions included making the environment more soothing and comforting, avoiding misuse of substances, and reaching out to people who could help one stay safe during crises. We found it particularly concerning that two of the reviewed apps provided explicit and detailed examples of means of suicide which the apps provided with the intention to help users remove access to said means. Such detailed examples could have an unintended provocative effect on some users and are therefore best avoided. Developers must pay close attention to the information provided in apps, mainly for technology-based suicide programs, considering that certain kinds of information can also have adverse or provocative effects [22].

Perspective-Shifting and Cognitive Strategies

Sixteen apps (37.20%) encouraged users to think through reasons for living, define, and focus on the meaning of hope for oneself, and to remind oneself of personal goals to help broaden one’s perspective at a time of crisis. Other suggestions targeting perspective-shifting included learning to challenge negative thoughts, recording a hope-instilling message for oneself to listen to at times of crises, and practicing positivity and gratitude. The latter typically falls under positive psychological interventions.

Calming/Soothing Strategies

Eleven apps (25.58%) recommended using calming/soothing strategies to help during times of crisis. These typically included prompts to practice deep breathing, mindfulness meditation, or other relaxation exercises or grounding strategies. Some apps included brief audio clips on different relaxation/mindfulness practices within the apps; however, only a few apps provided a rationale for these practices. A few apps provided examples such as listening to calming music, having a hot bath, painting, praying, journaling, or treating oneself to something small as calming or soothing activities while providing users space to list soothing activities per their needs. Exercise or walking was offered as a calming activity in some apps, as a distraction strategy in others.

Focusing and Problem-Solving Strategies

Seven apps (16.27%) included focusing and problem-solving strategies. These included suggestions of focusing on a few small problems first to reduce the sense of being overwhelmed, focusing on strategies that have helped in previous situations, and delaying making important decisions during times of crisis.

Self-Care and Well-Being

Seven apps (16.27%) highlighted self-care as an important strategy. These included suggestions such as regular and adequate nutrition, rest and physical activity, engaging in activities of interest and pleasure, and forming supportive relationships.

Motivational Strategies

Six apps (13.95%) included quotes on suicide prevention awareness or motivational statements to help users persist with or boost their efforts in dealing with a crisis. Some encouraged users to save inspiring photos, quotes, music, themes, and other resources that they could access during crises to build hope and motivation.

Observations and limitations

Several apps listed strategies as one-line generic suggestions -- for example, walking or exercise -- often without a clear rationale, making it challenging to group strategies into distinct categories. Failing to provide a rationale could reduce users’ motivation to apply skills [17], emphasizing the need for clear explanations.

While most apps facilitated access to crisis helplines, only a few included motivational elements beyond the disclaimer that encouraged users to call these helplines. Encouraging help-seeking behaviors is of immense importance, particularly in suicide prevention, where ambivalence and cognitive or emotional barriers could hinder help-seeking. Further, most of the apps identified through a Google Play Store search resulted in apps with suicide helpline numbers specific to the region where they were developed, most of them were not applicable to Indian audiences. Only a few included a directory of helpline numbers relevant for various countries. While suicide prevention content is relevant to audiences across the globe, helpline numbers are specific to countries. Only four indigenously developed apps were available in India, and two listed relevant helpline numbers but had no motivational elements to call helplines. They also did not include any additional resources such as emergency services in the user’s vicinity. Only one app provided evidence-based suicide prevention content; however, it failed to include strategies to manage suicidal thoughts or motivational elements. The inclusion of motivational elements for help-seeking is particularly important in apps aimed at Indian users, considering the stigma associated with mental illness and wide treatment gap.

App store search results -- unlike literature databases -- are dynamic, and the present study is limited in its generalizability as it provides an overview of apps available during a brief search period. Further, the review narrowly focused on a single app store most frequently used by Indian users and may have missed suicide prevention apps in other app markets. The review also specifically examined apps addressing suicide prevention exclusively and did not include apps on other mental health conditions providing suicide prevention content.

Implications and future directions

Suicide prevention apps such as those primarily intended to develop safety plans could serve as a helpful supplement to clinical care. However, considering that few apps have been empirically validated and some apps present potentially harmful content, clinicians need to scrutinize content before recommending them for use. If apps are to be used effectively in suicide prevention efforts for the general population, there is a need to develop and empirically validate apps that include motivational aspects to call helplines and provide evidence-based content. Specific to Indian users, there is also an urgent need for more indigenously developed apps that include suicide prevention content more relevant to the Indian population. This could include addressing barriers to help-seeking such as stigma, attitude towards suicide and mental illness, reducing family resistance by creating awareness, and equipping family members/significant others with skills to encourage help-seeking, along with relevant helplines.

Considering the relatively high prevalence of suicide in the younger, urban Indian population, where the use of smartphones is increasing, mobile apps could be utilized to supplement suicide prevention efforts by addressing some of the barriers to help-seeking. Making apps available in regional languages, in addition, could facilitate access to a wider population in India.

## Conclusions

The present review aimed to identify the nature of suicide prevention apps available to Indian Android phone users. We found numerous apps that mainly incorporated suicide prevention content, while a few included strategies to manage suicidal thoughts to varying degrees. Potentially harmful content such as implicit suggestions of self-harm was found in a small proportion of apps. The target audience ranged from individuals at risk of suicide to support providers, but a large proportion of apps failed to state the intended audience or scope of the app. Despite several apps being developed by individuals and institutes in the mental health field, only a small proportion provided supplementary information on evidence-based content or information on any research conducted on the app per se. These factors could make it difficult for a clinician or a potential user to identify a relevant app for use easily. While well-structured suicide prevention apps could supplement clinical care in some instances, clinicians need to scrutinize features and content before recommending an app for use. There is also an urgent need for indigenously developed apps with content more relevant to Indian users.
